# Prognostic Nomogram for Predicting Long-Term Overall Survival of Esophageal Cancer Patients Receiving Neoadjuvant Chemoradiotherapy Plus Surgery: A Population-Based Study

**DOI:** 10.3389/fsurg.2022.927457

**Published:** 2022-05-25

**Authors:** Mingduan Chen, Zhinuan Hong, Zhimin Shen, Lei Gao, Mingqiang Kang

**Affiliations:** ^1^ Department of Thoracic Surgery, Fujian Medical University Union Hospital, Fuzhou, China; ^2^ Key Laboratory of Cardio-Thoracic Surgery (Fujian Medical University), Fujian Province University, Fuzhou, China; ^3^ Key Laboratory of Ministry of Education for Gastrointestinal Cancer, Fujian Medical University, Fuzhou, China; ^4^ Fujian Key Laboratory of Tumor Microbiology, Fujian Medical University, Fuzhou, China

**Keywords:** esophageal cancer, neoadjuvant chemoradiotherapy, long-term survival, population-based study, SEER, follow-up plan

## Abstract

**Objective:**

Neoadjuvant chemoradiotherapy (nCRT) plays an important role in patients with locally advanced esophageal cancer (EC). We aim to determine the prognostic risk factors and establish a reliable nomogram to predict overall survival (OS) based on SEER population.

**Methods:**

Patients with EC coded by 04–15 in the SEER database were included. The data were divided into training group and verification group (7:3). The Cox proportional-risk model was evaluated by using the working characteristic curve (receiver operating characteristic curve, ROC) and the area under the curve (AUC), and a nomogram was constructed. The calibration curve was used to measure the consistency between the predicted and the actual results. Decision curve analysis (DCA) was used to evaluate its clinical value. The best cut-off value of nomogram score in OS was determined by using X-tile software, and the patients were divided into low-risk, medium-risk, and high-risk groups.

**Results:**

A total of 2,209 EC patients who underwent nCRT were included in further analysis, including 1,549 in the training cohort and 660 in the validation group. By Cox analysis, sex, marital status, T stage, N stage, M stage, and pathological grade were identified as risk factors. A nomogram survival prediction model was established to predict the 36-, 60-, and 84-month survival. The ROC curve and AUC showed that the model had good discrimination ability. The correction curve was in good agreement with the prediction results. DCA further proved the effective clinical value of the nomogram model. The results of X-tile analysis showed that the long-term prognosis of patients in the low-risk subgroup was better in the training cohort and the validation cohort (*p* < 0.001).

**Conclusion:**

This study established an easy-to-use nomogram risk prediction model consisting of independent prognostic factors in EC patients receiving nCRT, helping to stratify risk, identify high-risk patients, and provide personalized treatment options.

## Introduction

Esophageal cancer (EC) is one of the most common and challenging types of cancer, and many patients with EC are found to be locally advanced at first visit (1). The prognosis of patients with locally advanced EC after surgery alone is poor, and the 5-year survival rate is only 25% (2).

The pattern of nCRT, combined with radical esophagectomy, can improve long-term survival among patients with locally advanced EC (3, 4). In the NEOCRTEC5010 trial, compared with surgery-alone groups, nCRT plus surgery had a significantly longer median overall survival (OS) (100.1 months vs. 66.5 months) and disease-free survival (100.1 months vs. 41.7 months) (3). Recently, Eyck et al. noted that compared with surgery alone, the absolute benefit of nCRT plus surgery in 10-year OS was 13% (38% vs. 25%), with a hazard ratio of 0.60 (95% CI, 0.46–0.80) (4). Based on present evidence, nCRT, combined with radical esophagectomy, is recommended as the standard treatment for locally advanced EC.

The Union for International Cancer Control Tumor/Node/Metastasis (TNM) staging system is a common and widely used tool to predict the prognosis and instruct in adjuvant therapy of EC. However, sometimes the TNM staging system is not accurate enough, and the clinical survival results of patients with similar TNM staging may be inconsistent (5–8). Thus, it is necessary to establish a patient prediction model including other prognostic factors after nCRT in order to make a more accurate prediction. Considering the fact that the case number in previous reports was limited, we aimed to find the independent prognostic factors in EC patients receiving nCRT plus surgery based on a large population, which could contribute to risk stratification and help clinicians identify patients with high risk and provide personalized treatment options.

## Methods

### Patient Selection

This population-based retrospective study used data from the SEER database. The data used in this study were downloaded from SEER * stat software (version 8.3.6). Our study included patients with EC who underwent nCRT plus surgery between the years 2004 and 2015. The inclusion criteria were as follows: (1) patients with primary EC, (2) patients who received nCRT, and (3) patients with adequate clinicopathological characteristics, demographic data, and follow-up information. Patients with autopsy confirmation or dead were excluded. Finally, among 44,457 EC patients, 2,209 EC patients who underwent nCRT plus esophagectomy were selected to make up the study cohort.

### Definition of Variables

Patients’ demographic characteristics (age, sex, race, insurance status, and marital status), disease characteristics (histology, primary site, tumor size, grade, T, N, and M stages), treatment modalities (radiotherapy, chemotherapy), duration of survival, and life status were included for analysis in this study. Using X-tile software (Yale University, New Haven, CT, USA) to determine the optimal cut-off value for age in OS, the patients were divided into three groups (<50, 50–65, and > 65 years). We divided patients into three groups (<51, 51–76, and >76 mm) based on tumor size. The primary site was defined according to the International Classification of Diseases in Oncology code: lower third of the esophagus (15.5), middle third of the esophagus (15.4), upper third of the esophagus (15.3), and others. The histological types of patients were adenocarcinoma, squamous cell carcinoma, and others. Tumor differentiation was divided into grade I, II, III, and IV groups. All cases were staged by the 7th edition TNM staging system.

### Statistical Analysis

The primary endpoint of the study was OS, defined as the time between the date of diagnosis and the date of death from any cause or the date of last follow-up. First, the patients were randomly divided into training and validation groups at a ratio of 7:3 in R software. Univariate and multivariate Cox proportional hazards regression analyses were used to identify independent prognostic factors in the training cohort. Nomograms based on independent risk factors were built to predict patients’ OS. The receiver operating characteristic curve (ROC) and area under the curve (AUC) were used to evaluate the recognition ability of the nomogram model. Calibration curves were used to measure the agreement between predicted and actual results. Decision curve analysis (DCA) was used to evaluate its clinical application. X-tile software was used to determine the optimal cut-off value of the nomogram OS score to classify patients into low-, medium-, and high-risk groups. To further validate the accuracy and validity of the nomogram model, we performed an evaluation of the model’s validity in the validation set. R software (version 3.6.1) was used for statistical analysis. A two-sided *p* < 0.05 was considered statistically significant.

## Results

### Baseline Characteristics of Esophageal Cancer Receiving Neoadjuvant Chemoradiotherapy Plus in the Training Cohort and Validation Group

**Figure 1:**
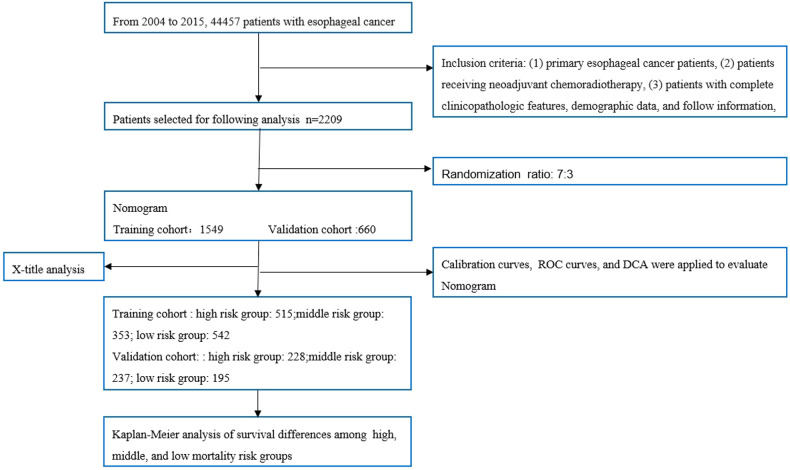
Flowchart of patient selection and analysis procedures. ROC, receiver operating characteristic; DCA, decision curve analysis.

The patient screening flow chart is shown in Figure 1. From the years 2004 to 2015, there were 44,457 patients with EC in the SEER database, of which 2,209 met our study criteria. We randomized 2,209 patients in a 7:3 ratio into a training cohort (1,549 patients) and a validation cohort (660 patients). The age of most patients ranged between 50 and 65 years at first visit, with 837 (54.03%) in the training group and 366 in the validation group (55.45%). The majority of histology type was adenocarcinoma, with 1,094 (70.62%) in the training group and 475 (71.97%) in the validation group. There were no statistically significant differences between the training cohort and the validation cohort in baseline characteristics such as age, grade, T stage, N stage, chemotherapy, insurance status, histological type, radiotherapy, and marital status (*p* < 0.05). Comparisons of baseline characteristics between the two groups are summarized in Table 1.

**Table 1:** Comparisons of demographic and clinicopathological characteristics in the training cohort and validation cohort.

Variables	Training cohort (*n* = 1,549)	Validation cohort (*n* = 660)	*p*-Value
Age			0.74
<50	155	68	
50–65	837	366	
>65	557	226	
Sex			0.65
Female	237	96	
Male	1,312	564	
Marital status			0.12
Married	1,080	482	
Unmarried	469	178	
Race			0.10
Black	86	28	
Other	51	32	
White	1,412	600	
Histology			0.70
Adenocarcinoma	1,094	475	
Squamous cell carcinoma	286	112	
Other	169	73	
T stage			0.90
T1-2	417	176	
T3-4	1,132	484	
N stage			0.49
N0	521	232	
N1	1,028	428	
M stage			0.84
M0	1,404	600	
M1	145	60	
Grade			0.44
Grade I	76	33	
Grade II	658	301	
Grade III	793	320	
Grade IV	22	6	
Primary site			0.37
Upper	14	9	
Middle	169	60	
Lower	1,233	540	
Other	133	51	
Tumor size			0.89
<51 mm	1,008	435	
51–76 mm	330	140	
>76 mm	211	85	
Radiotherapy after surgery			0.84
With	56	25	
Without	1,493	635	
Chemotherapy after surgery			0.57
With	134	62	
Without	1,415	598	

### Results of Univariate and Multivariate Cox Analysis in the Training Cohort

Patients included in the analysis were randomized in a 7:3 ratio, with 1,549 patients in the training cohort and 660 patients in the validation cohort. We used the training cohort to determine prognostic risk factors. Multivariate COX analysis noted that total six factors (M stage, N stage, T stage, differentiation grade, sex, and marital status) were identified as independent prognostic risk factors. The results of univariate and multivariate Cox analyses of the training cohort are summarized in Table 2.

**Table 2:** Univariate and multivariate Cox analysis of overall survival for esophageal cancer patients receiving neoadjuvant therapy plus surgery in the training cohort.

Characteristics	Univariate Cox analysis		Multivariate Cox analysis	
	HR 95%CI	*p*	HR 95%CI	*p*
Age				
<50	1.00			
50–65	1.1 (0.89–1.36)	0.40		
>65	1.18 (0.95–1.47)	0.14		
Sex				
Female	1.00			
Male	1.21 (1.02–1.44)	0.03	1.21 (1.02–1.44)	0.03
Race				
Black	1.00			
White	0.97 (0.75–1.25)	0.82		
Other	0.73 (0.47–1.14)	0.17		
Marital	1.00			
Married				
Unmarried	1.17 (1.03–1.33)	0.02	1.2 (1.06–1.37)	0.01
Grade				
Grade I	1.00		1.00	
Grade II	1.27 (0.93–1.73)	0.14	1.23 (0.9–1.67)	0.20
Grade III	1.54 (1.13–2.09)	0.01	1.46 (1.08–1.99)	0.01
Grade IV	2.01 (1.13–3.56)	0.02	1.91 (1.07–3.38)	0.03
Histology				
Adenocarcinoma	1.00			
Squamous cell carcinoma	0.9 (0.74–1.11)	0.34		
Other	1.2 (0.95–1.5)	0.13		
M stage				
M0	1.00		1.00	
M1	1.34 (1.1–1.63)	<0.001	1.31 (1.08–1.59)	0.01
N stage				
N0	1.00		1.00	
N1	1.56 (1.32–1.84)	<0.001	1.51 (1.28–1.78)	<0.001
Primary site				
Upper	1.00			
Middle	1.05 (0.53–2.06)	0.74		
Lower	1.12 (0.58–2.15)	0.90		
Other	1.43 (0.72–2.83)	0.29		
T stage				
T1-2	1.00			
T3-4	1.33 (1.16–1.53)	<0.001	1.22 (1.06–1.41)	0.01
Tumor size				
<51 mm	1.00			
51–76 mm	1.07 (0.92–1.24)	0.39		
>76 mm	1.18 (0.99–1.4)	0.07		
Radiotherapy after surgery				
Without	1.00			
With	1.09 (0.79–1.49)	0.60		
Chemotherapy after surgery				
With	1.00			
Without	0.93 (0.75–1.14)	0.47		

### Development and Validation of a Prognostic Nomogram

**Figure 2:**
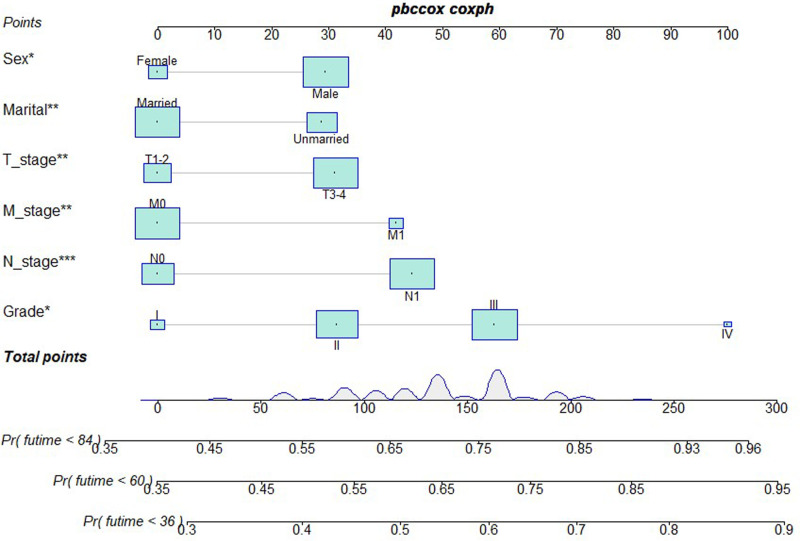
Nomogram model to predict the OS at 36-, 60-, and 84 months in patients receiving neoadjuvant chemoradiotherapy plus surgery.

**Figure 3:**
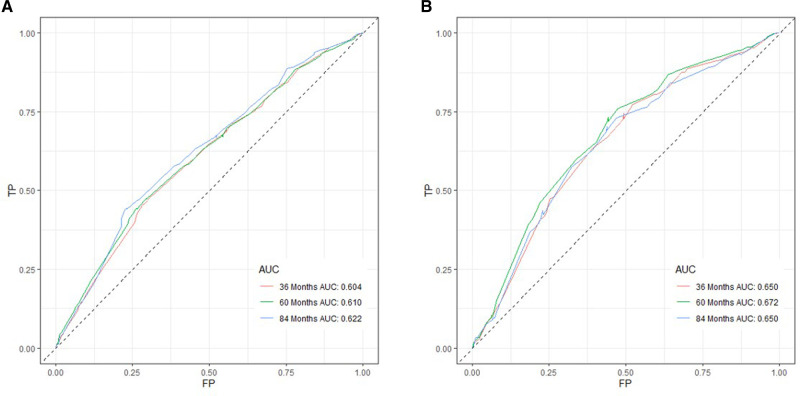
ROC curves for survival prediction of patients receiving neoadjuvant therapy plus surgery. (**A**) ROC curves of 36-, 60-, and 84 months in the training cohort, (**B**) ROC curves of 36-, 60-, and 84 months in the validation cohort. TP, true positive rate; FP, false positive rate; ROC, receiver operating characteristic.

**Figure 4:**
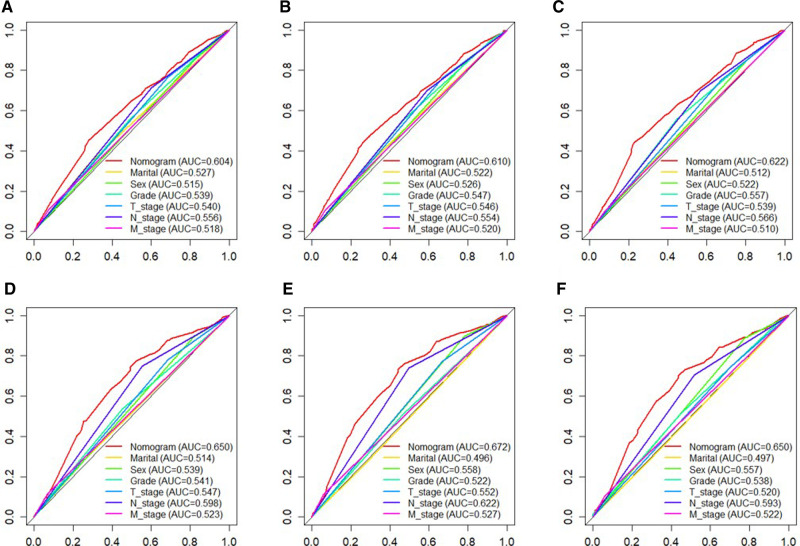
The ROC curves of nomogram and all independent predictors at 36- (**A**), 60- (**B**), and 84 months (**C**) in the training cohort and at 36- (**D**), 60- (**E**), and 84 months (**F**) in the validation cohort. ROC, receiver operating characteristic.

**Figure 5:**
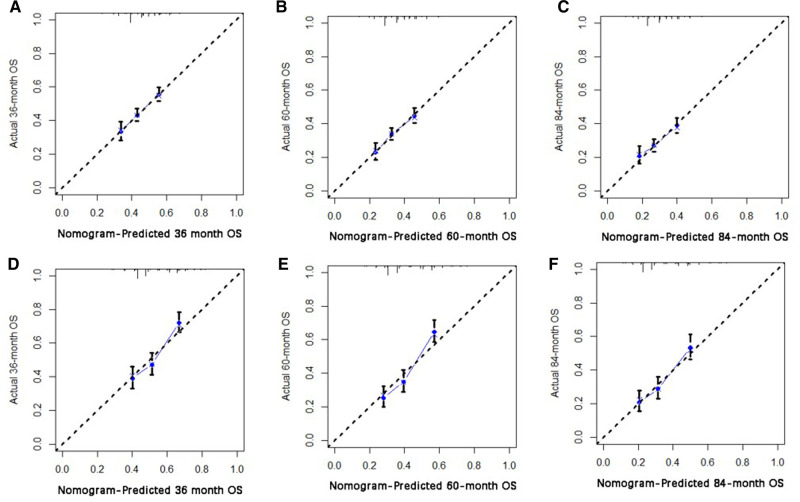
The calibration curve for predicting patient survival at (**A**) 36 months, (**B**) 60 months, (**C**) and 84 months in the training cohort, and at (**D**) 36 months, (**E**) 60 months, (**F**), and 84 months in the validation cohort. The nomogram-predicted probability of the overall survival rate is plotted on the X-axis, and the actual overall survival rate is plotted on the Y-axis.

**Figure 6:**
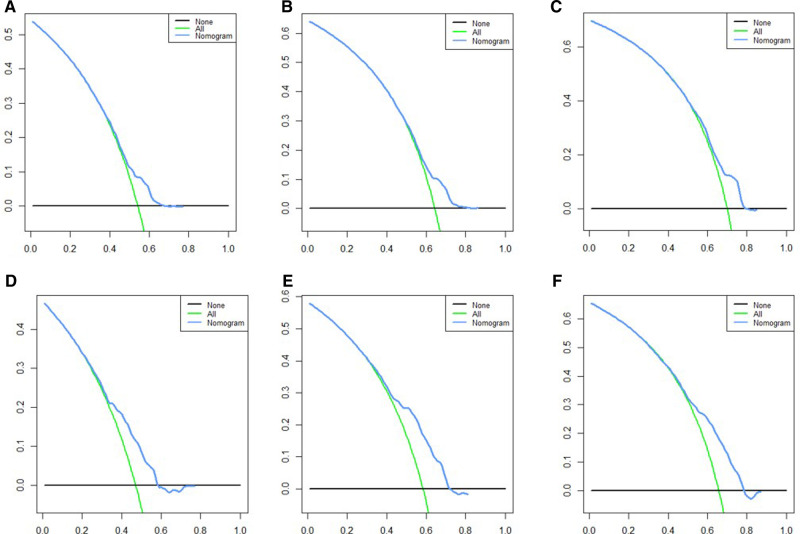
DCA for survival prediction. (**A**) DCA of 36 months in the training cohort, (**B**) DCA of 60 months in the training cohort, (**C**) DCA of 84 months in the training cohort, (**D**) DCA of 36 months in the validation cohort, (**E**) DCA of 60 months in the validation cohort, (**F**) DCA of 84 months in the validation cohort. DCA, decision curve analysis.

Based on independent prognostic risk factors, we established a nomogram model to predict 36-, 60-, and 84-month OS (Figure 2). Time-dependent ROCs noted that this model not only performed well in predicting OS in both training cohort and validation cohort (Figure 3), but also had a higher prediction accuracy than individual prognostic factors in both cohorts (Figure 4). The calibration curves indicated that the predicted results of this model were highly consistent with the actual results in both cohorts (Figure 5). DCA also proved that this model had strong clinical applicability in both cohorts (Figure 6).

### Risk Stratification Based on Nomogram Score and Kaplan–Meier Curves for Overall Survival

**Figure 7:**
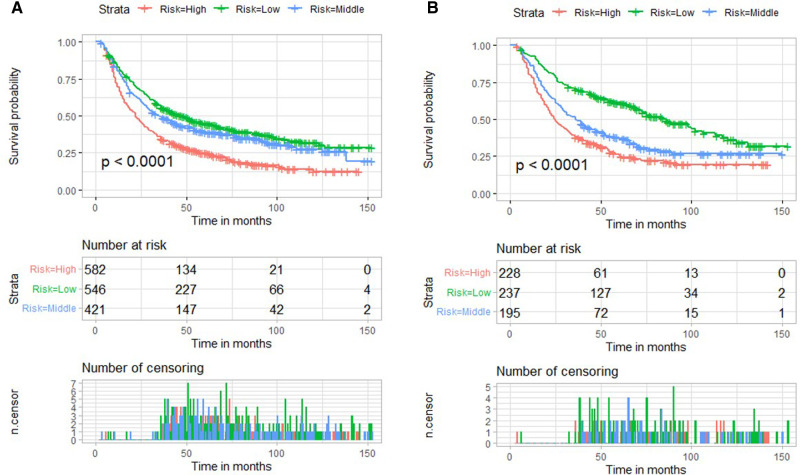
Risk stratification based on nomogram score and Kaplan–Meier curves for overall survival in the training cohort (**A**) and validation cohort (**B**).

We used X-tile software to determine the cut-off values of nomogram score and divided patients into low-, medium-risk subgroups, and high-risk subgroups. The low-risk subgroup was defined as that which had a nomogram score <22, and the high-risk subgroup was defined as that which had a nomogram score >36. Patients with a nomogram score between 22 and 36 were divided into medium-risk subgroup. Interestingly, compared with the high-risk subgroup, we noted that patients stratified as low risk had a better survival rate in both validation cohort and training cohort (*p* < 0.001) **(**Figure 7**)**.

## Discussion

To our knowledge, this was the first study focusing on the prognostic risk factors of EC receiving nCRT plus surgery based on a population analysis. We found that T stage, N stage, M stage, pathological grade, sex, and marital status were independent risk factors for poor OS. A reliable prognostic nomogram was established. Further, we also found that patients in the high-risk group had a poorer prognosis (*p* < 0.001) in both training and validation cohorts. For patients with high risk, a more active and frequent follow-up plan is necessary.

Based on the latest AJCC staging system (the 8th edition), pathological grade is not a staging factor for patients receiving neoadjuvant therapy. However, we found that the pathological grade was an independent risk factor for OS among patients receiving nCRT plus surgery, which was consistent with that of other reports (9, 10). He et al. noted that EC patients with poorly differentiated tumors respond better to nCRT than those with well-differentiated or moderately differentiated tumors; however, they have extremely poor long-term survival rates (10). One possible reason is that due to the high turnover, poorly differentiated tumors have a higher probability of being affected by DNA damage and apoptosis following nCRT, which contributes to a higher pathological response (10). For EC patients with the same pathological stage, a worse pathological grade often indicates a worse prognosis and a higher postoperative recurrence rate (11). Poorly differentiated EC had a worse prognosis due to the higher rate of lymph node metastasis and distant organ metastasis rate (such as liver metastasis) (12). Further, the pathological grade could also guide adjuvant therapy after neoadjuvant therapy (12, 13).

We found that the status of being unmarried was an independent risk factor for poor OS. Lan et al. noted that unmarried status was an independent risk factor for cancer specific death (CSS) in men with esophageal adenocarcinoma (EAC) (HR: 1.11; 95% CI: 1.04 –1.18; *p* = 0.001) (14). Zhou et al. noted that unmarried status was an independent prognostic factor for gastric neuroendocrine neoplasms, with an HR of 1.56 for OS and an HR of 1.33 for CSS (15). Chen et al. reported that unmarried status was significantly associated with decreased CSS in non-small cell lung cancer, with an HR of 1.142 (16). We attributed the difference in OS between married and unmarried statuses to the following reasons: First, the unmarried population has less financial support from spouses, which may result in less effective treatment such as surgery, chemotherapy, and radiotherapy. This may be partly explained by underinsurance and a lack of financial support (17). Second, unmarried patients are more likely to exhibit unhealthy behaviors such as smoking, drinking, and high-fat diet (18). Among unmarried individuals without spouse supervision or reminders, more individuals do not participate in routine cancer screening. Third, being unmarried is associated with psychiatric disorders such as anxiety and depression. The loss of emotional and psychological support from spouses may expose unmarried patients to the risk of adverse outcomes (19). Moreover, in psychoneuroimmunology, stress and depression can promote immune dysfunction and the progression of various cancers by activating the adrenal axis (20). Thus, to improve the long-term survival rate of the unmarried population, providing appropriate psychosocial support and appropriate financial assistance may be helpful. From a surgeon’s point of view, regular follow-up visits should be conducted for the unmarried population.

We found that male gender was another independent risk factor for poor prognosis. Previous studies suggested a better prognosis in women than in men with EC. Kauppila et al. suggested that the prognosis of ESCC resection in women seemed to be better than that in men, especially for early-stage tumors, whereas the prognosis of EAC did not differ in sex (21). Bohanes et al. reported that women with locally advanced EC (55 years old) had significantly better outcomes than men (22). The possible mechanisms of difference between males and females were as follows: First, the comorbidity score in males was higher than that in females (23); however, there was no related data in the SEER database. Second, estrogen receptors (ERs) are highly expressed in ESCC, and estrogens were confirmed to inhibit squamous cell tumor growth (24, 25). Third, oncogenic types of HPV have been proved to play an important role in ESCC in high-risk areas (26, 27).

To our best knowledge, research based on population for EC patients receiving nCRT is relatively limited. In this context, this study established a highly reliable model to predict 36-, 60-, and 84-month OS for EC patients receiving nCRT. However, our study has the following three limitations. First, it has the limitation of retrospective nature. We attempted to avoid potential bias through rigorous patient selection. Second, the chemoradiotherapy regimens were not recorded in detail in the SEER database. Radiotherapy or chemotherapy doses described in the SEER data are categorized as yes or no/unknown. All studies using the seer dataset cannot avoid common shortcomings. A comparison of the efficacy of different treatment regimens was beyond the scope of this study. Third, nCRT had limited application in our hospital due to the difficulties faced in the issue of quality control of radiotherapy and the model being validated internally in the SEER population. Neoadjuvant chemotherapy (nCT) is more popular in Asian countries, especially in China and Japan. Whether this nomogram is suitable for patients receiving neoadjuvant chemotherapy or surgery alone remains unclear. Therefore, we look forward to the establishment of a database based on Asian populations of ESCC patients.

## Conclusion

This study established an easy-to-use nomogram risk prediction model consisting of independent prognostic factors in EC patients receiving nCRT, helping to stratify risk, identify high-risk patients, and provide personalized treatment options.

## Data Availability

The raw data supporting the conclusions of this article will be made available by the authors, without undue reservation.

## References

[1] Lu L Mullins CS Schafmayer C Zeißig S Linnebacher M. A global assessment of recent trends in gastrointestinal cancer and lifestyle-associated risk factors. Cancer Commun (Lond). (2021) 41(11):1137–51. 10.1002/cac2.12220 34563100 PMC8626600

[2] Herskovic A Russell W Liptay M Fidler MJ Al-Sarraf M. Esophageal carcinoma advances in treatment results for locally advanced disease: review. Ann Oncol. (2012) 23(5):1095–103. 10.1093/annonc/mdr433 22003242

[3] Yang H Liu H Chen Y Zhu C Fang W Yu Z, Neoadjuvant chemoradiotherapy followed by surgery versus surgery alone for locally advanced squamous cell carcinoma of the esophagus (NEOCRTEC5010): a phase III multicenter, randomized, open-label clinical trial. J Clin Oncol. (2018) 36(27):2796–803. 10.1200/JCO.2018.79.1483. 30089078 PMC6145832

[4] Eyck BM van Lanschot JJB Hulshof MCCM van der Wilk BJ Shapiro J van Hagen P Ten-year outcome of neoadjuvant chemoradiotherapy plus surgery for esophageal cancer: the randomized controlled CROSS trial. J Clin Oncol. (2021) 39(18):1995–2004. 10.1200/JCO.20.03614 33891478

[5] Li Y Lu Z Che Y Wang J Sun S Huang J Immune signature profiling identified predictive and prognostic factors for esophageal squamous cell carcinoma. Oncoimmunology. (2017) 6(11):e1356147. 10.1080/2162402X.2017.1356147 29147607 PMC5674952

[6] Ding T Liu C Huang B Chu L Wei L Lin Y A survival prediction nomogram for esophageal squamous cell carcinoma treated with neoadjuvant chemoradiotherapy followed by surgery. Cancer Manag Res. (2021) 13:7771–82. 10.2147/CMAR.S329687 34675672 PMC8519412

[7] Li J Mei X Sun D Guo M Xie M Chen X. A nutrition and inflammation-related nomogram to predict overall survival in surgically resected esophageal squamous cell carcinoma (ESCC) patients. Nutr Cancer. (2022) 74(5):1625–35. 10.1080/01635581.2021.1957131 34369223

[8] Huang GW Xue YJ Wu ZY Xu XE Wu JY Cao HH A three-lncRNA signature predicts overall survival and disease-free survival in patients with esophageal squamous cell carcinoma. BMC Cancer. (2018) 18(1):147. 10.1186/s12885-018-4058-6 29409459 PMC5801805

[9] Merritt RE Abdel-Rasoul M Souza DM Kneuertz PJ. Nomograms for predicting overall and recurrence-free survival after trimodality therapy for esophageal adenocarcinoma. J Surg Oncol. (2021) 123(4):881–90. 10.1002/jso.26349 33333590

[10] He W Mao T Yan J Leng X Deng X Xie Q Moderately differentiated esophageal squamous cell carcinoma has a poor prognosis after neoadjuvant chemoradiotherapy. Ann Transl Med. (2021) 9(8):706. 10.21037/atm-21-1815 33987404 PMC8106115

[11] Wang Y Xiao P Yang N Wang X Ma K Wu L Unresected small lymph node assessment predicts prognosis for patients with pT3N0M0 thoracic esophageal squamous cell carcinoma. World J Surg Oncol. (2021) 19(1):303. 10.1186/s12957-021-02412-1 34657600 PMC8522218

[12] Zhang S Guo J Zhang H Li H Hassan MOO Zhang L. Metastasis pattern and prognosis in men with esophageal cancer patients: a SEER-based study. Medicine (Baltimore). (2021) 100(25):e26496. 10.1097/MD.0000000000026496 34160464 PMC8238299

[13] Li H Zhang S Guo J Zhang L. Hepatic metastasis in newly diagnosed esophageal cancer: a population-based study. Front Oncol. (2021) 11:644860. 10.3389/fonc.2021.644860 34041021 PMC8143266

[14] Lan T Liu W Lu Y Zheng R Luo H Shao X Role of marital status on the prognosis in esophagus adenocarcinoma: a real-world competing risk analysis. Future Oncol. (2020) 16(35):2923–37. 10.2217/fon-2020-0613 32892636

[15] Zhou YJ Lu XF Zheng KI Wang QW Chen JN Zhang QW Marital status, an independent predictor for survival of gastric neuroendocrine neoplasm patients: a SEER database analysis. BMC Endocr Disord. (2020) 20(1):111. 10.1186/s12902-020-00565-w 32703291 PMC7376955

[16] Chen Z Yin K Zheng D Gu J Luo J Wang S Marital status independently predicts non-small cell lung cancer survival: a propensity-adjusted SEER database analysis. J Cancer Res Clin Oncol. (2020) 146(1):67–74. 10.1007/s00432-019-03084-x 31786738 PMC11804430

[17] Levitz NR Haji-Jama S Munro T Gorey KM Luginaah IN Bartfay E Multiplicative disadvantage of being an unmarried and inadequately insured woman living in poverty with colon cancer: historical cohort exploration in California. BMC Womens Health. (2015) 15:8. 10.1186/s12905-015-0166-5 25783640 PMC4333264

[18] Kim A Lee JA Park HS. Health behaviors and illness according to marital status in middle-aged Koreans. J Public Health (Oxf). (2018) 40(2):e99–e106. 10.1093/pubmed/fdx071 30020525

[19] Aizer AA Chen MH McCarthy EP Mendu ML Koo S Wilhite TJ Marital status and survival in patients with cancer. J Clin Oncol. (2013) 31(31):3869–76. 10.1200/JCO.2013.49.6489 24062405 PMC4878087

[20] Reiche EM Morimoto HK Nunes SM. Stress and depression-induced immune dysfunction: implications for the development and progression of cancer. Int Rev Psychiatry. (2005) 17(6):515–27. 10.1080/02646830500382102 16401550

[21] Kauppila JH Wahlin K Lagergren P Lagergren J. Sex differences in the prognosis after surgery for esophageal squamous cell carcinoma and adenocarcinoma. Int J Cancer. (2019) 144(6):1284–91. 10.1002/ijc.31840 30168595

[22] Bohanes P Yang D Chhibar RS Labonte MJ Winder T Ning Y Influence of sex on the survival of patients with esophageal cancer. J Clin Oncol. (2012) 30(18):2265–72. 10.1200/JCO.2011.38.8751 22585694 PMC3397720

[23] Backemar L Lagergren P Johar A Lagergren J. Impact of co-morbidity on mortality after oesophageal cancer surgery. Br J Surg. (2015) 102(9):1097–105. 10.1002/bjs.9854 26059747

[24] Xie SH Santoni G Lagergren J. Menopausal hormone therapy and risk of oesophageal adenocarcinoma in a population-based cohort study. Br J Cancer. (2022) 126(1):129–33. 10.1038/s41416-021-01575-8 34671128 PMC8727583

[25] Pinton G Manzotti B Balzano C Moro L. Expression and clinical implications of estrogen receptors in thoracic malignancies: a narrative review. J Thorac Dis. (2021) 13(3):1851–63. 10.21037/jtd-20-2277 33841973 PMC8024832

[26] Syrjänen KJ. HPV infections and oesophageal cancer. J Clin Pathol. (2002) 55:721–8. 10.1136/jcp.55.10.721 12354793 PMC1769774

[27] Tribius S Ihloff AS Rieckmann T Petersen C Hoffmann M. Impact of HPV status on treatment of squamous cell cancer of the oropharynx: what we know and what we need to know. Cancer Lett. (2011) 304(2):71–9. 10.1016/j.canlet.2011.02.002 21376458

